# Book review: Transcranial direct current stimulation in neuropsychiatric disorders. Clinical principles and management

**DOI:** 10.3389/fnins.2022.1082143

**Published:** 2022-11-24

**Authors:** Eugenia Gianni, Franca Tecchio

**Affiliations:** ^1^Laboratory of Electrophysiology for Translational NeuroScience (LET'S), Institute of Cognitive Sciences and Technologies—Consiglio Nazionale delle Ricerche, Rome, Italy; ^2^Unit of Neurology, Neurophysiology, Neurobiology, Department of Medicine, Università Campus Bio-Medico di Roma, Rome, Italy; ^3^Faculty of Psychology, Uninettuno University, Rome, Italy

**Keywords:** transcranial electric stimulation (tES), transcranial direct current stimulation (tDCS), neuropsychiatric disorders, neuromodulation, safety, accessibility

## Summary of the book

The book, first released in 2016 (Brunoni et al., 2016) and then re-issued with a novel expanded edition in 2021 (Brunoni et al., 2021), is rather complete, touching several aspects of the use of transcranial direct current stimulation (tDCS), and well-structured, dividing itself into five parts.

### Part I: Introduction and mechanisms of action

By the very beginning the reader is immersed into an interesting historical overview of the use of electrical stimulation to cure ailments. From electric fish to the most modern applications of tDCS the reader may learn that the use of electricity to cure illness has a long tradition and deep roots in the history of medicine. Further, the reader has the possibility to be acquainted with the physiology, neural mechanisms, and computational models of tDCS, transcranial alternating current stimulation (tACS), and transcranial random noise stimulation (tRNS). Moreover, he/she is offered the results of transcranial electric stimulation (tES) related investigations throughout animal models: how can these teach us about the biomarkers of tDCS and the other techniques?

### Part II: Research methods

Going further, the reader may learn that to properly study mechanisms, targets and effects of tDCS and repetitive transcranial magnetic stimulation (rTMS) it is possible to combine them with neuroimaging techniques: from electroencephalography (EEG) to magnetic resonance imaging (MRI) and functional magnetic resonance imaging (fMRI). Not only we can closely study tES mechanisms and effects and thus finely modulate the target accordingly when tES is applied to the scalp but also when applied to the cerebellum and spinal cord. How to optimize tES experimental designs is the last focus of this methodological part.

### Part III: tDCS in the life cycle

Physiological mechanisms and effects of tDCS are presented also in the developing brain and in mothers in the perinatal period. In healthy young adults modulating high level cognitive functioning is useful both for exploring novel hypothesis on brain functioning and for improving cognitive performance as well as it is possible to enhance exercise motor performance. Unraveling the underpinnings of cognitive and behavioral processes in healthy subjects is the relevant aim of applying tDCS in the fields of social and emotional research. Finally, interesting results are emerging from the application of tDCS on neurodegenerative diseases like Alzheimer's, but further and wider studies are required.

### Part IV: Applications of tDCS in neuropsychiatric disorders

In the field of mood disorders, where the drug refractoriness is high, we learn that tDCS can be a valid therapeutic alternative, but further research is to be carried out to formulate proper predictive models. Promising results were found also in the fields of schizophrenia in the relieve of both hallucinations and negative symptoms for which, though, further clinical trials are required. The same holds for obsessive compulsive disorder (OCD), anxiety disorders, Post-traumatic stress disorder (PTSD), and attention deficit hyperactivity disorder (ADHD). Beneficial effects were found for the use of tDCS in substance-abuse disorders while research on the effects of tDCS on cognitive functions in various disorders offered discrepant results. Effectiveness of tDCS was shown for epilepsy and pain syndromes. Encouraging, pioneering results were found for tinnitus and disorders of consciousness. Finally, positive effects of tDCS on neuroplasticity emphasize its promise in the framework of rehabilitation.

### Part V: The clinical use of tDCS

Beyond these promising results, what is it possible to say in terms of safety and tolerability of tDCS? Evidence of minimal adverse events is strongly supported. Safety, accessibility, and convenience in terms of costs makes tDCS an optimal treatment also for home-environment. However, it is properly the extreme accessibility of tDCS that makes important to consider ethical aspects related to misuse and autonomy and to reflect upon defining regulatory aspects and invite caution. Finally, effects and limitations of combination of tDCS with pharmacotherapy and psychotherapy or neurobehavioral interventions are discussed.

## Evaluation of the book content

From the mechanisms of action of tDCS, tRNS, and tACS, to the relevant methodological aspects of tDCS and rTMS applications, to the effects of tDCS across development in healthy and patients' brains and in neuropsychiatric disorders to safety-related, ethical, and regulatory aspects, the book offers a 360-degree well-integrated overview of current and future perspective in the use of tDCS without avoiding speaking of its most proximal techniques such as tACS, tRNS, and rTMS. In doing so, it indicates, with accuracy and detail both the innovative and progressive aspects of current studies involving the application of these techniques as well as their current limitations.

## Discussion of the book's content in light of the current needs of the community

“Why writing a book on transcranial direct current stimulation?” we ask ourselves together with the Professor of Psychiatry and Psychotherapy in the foreword to the First Edition of the book, given that we are provided with a great variety of open-access scientific data and articles on this topic. Sharing his point of view, nowadays it acquires importance to provide a critical point of view on a scientific technique with relevant applications to real life in the face of the temptation of most people to fall into false beliefs being easily induced to a do-it-yourself use of the tDCS. On the other side we think it is extremely important to offer a broad and comprehensive overview that helps highlight the big potential and novelty of tES technologies [belonging to the broader field of electroceuticals (Reardon, 2014)] and to stress how they can constitute alternative approaches to pharmacological therapies, providing interventions that are able to safely alleviate annoying symptoms while decreasing costs and side effects (Lefaucheur et al., 2017). Notably, in a recent quantitative review, we showed how is it possible to include tDCS treatments in the framework of medical therapies under the indications of the international authorities classifying them between moderately and highly recommendable (Gianni et al., 2021).

## Author contributions

EG wrote the commentary with the supervision of FT. Both authors contributed to the article and approved the submitted version.

## Conflict of interest

The authors declare that the research was conducted in the absence of any commercial or financial relationships that could be construed as a potential conflict of interest.

## Publisher's note

All claims expressed in this article are solely those of the authors and do not necessarily represent those of their affiliated organizations, or those of the publisher, the editors and the reviewers. Any product that may be evaluated in this article, or claim that may be made by its manufacturer, is not guaranteed or endorsed by the publisher.
